# Impact of ApoE Polymorphism and Physical Activity on Plasma Antioxidant Capability and Erythrocyte Membranes

**DOI:** 10.3390/antiox8110538

**Published:** 2019-11-09

**Authors:** Rebecca Piccarducci, Simona Daniele, Jonathan Fusi, Lucia Chico, Filippo Baldacci, Gabriele Siciliano, Ubaldo Bonuccelli, Ferdinando Franzoni, Claudia Martini

**Affiliations:** 1 Department of Pharmacy, University of Pisa, 56126 Pisa, Italy; rebecca.piccarducci@farm.unipi.it (R.P.); simona.daniele@unipi.it (S.D.); 2 Department of Clinical and Experimental Medicine, University of Pisa, 56100 Pisa, Italy; jonathan.fusi@gmail.com (J.F.); lucia.chico@med.unipi.it (L.C.); filippo.baldacci@unipi.it (F.B.); gabriele.siciliano@unipi.it (G.S.); ubaldo.bonuccelli@unipi.it (U.B.)

**Keywords:** ApoE ε4, physical activity, erythrocytes, oxidative stress, cellular membrane, amyloid beta

## Abstract

The allele epsilon 4 (ε4) of apolipoprotein E (ApoE) is the strongest genetic risk factor for Alzheimer’s disease (AD). ApoE protein plays a pivotal role in the synthesis and metabolism of amyloid beta (Aβ), the major component of the extracellular plaques that constitute AD pathological hallmarks. Regular exercise is an important preventive/therapeutic tool in aging and AD. Nevertheless, the impact of physical exercise on the well-being of erythrocytes, a good model of oxidative stress and neurodegenerative processes, remains to be investigated, particularly depending on ApoE polymorphism. Herein, we evaluate the oxidative status, Aβ levels, and the membrane’s composition of erythrocytes in a cohort of human subjects. In our hands, the plasma antioxidant capability (AOC), erythrocytes membrane fluidity, and the amount of phosphatidylcholine (PC) were demonstrated to be significantly decreased in the ApoE ε4 genotype and non-active subjects. In contrast, erythrocyte Aβ content and lipid peroxidation increased in ε4 carriers. Regular physical exercise was associated with an increased plasma AOC and membrane fluidity, as well as to a reduced amount of erythrocytes Aβ. Altogether, these data highlight the influence of the ApoE genotype on erythrocytes’ well-being and confirm the positive impact of regular physical exercise.

## 1. Introduction

The apolipoprotein E (ApoE) is a 299 amino acid glycoprotein encoded by the ApoE gene, and existing in three polymorphic alleles. Consequently, the human ApoE protein occurs in three ApoE isoform, ε2, ε3, and ε4, which present a global frequent of 8.4%, 77.7%, and 13.7%, respectively. These isoforms differ from each other for the amino acids at positions 112 and 158 that alter the structure and, subsequently, the function of each isoform [1,2].

ApoE mediates lipid transport and contributes to liver cholesterol metabolism in an isoform-dependent manner, and has been linked to hyperlipidemia and hypercholesterolemia [2,3]. In the central nervous system, ApoE is primarily released by astrocytes and is involved in cholesterol transport to neuronal cells via ApoE receptors [2].

Furthermore, in the last decades, ApoE protein has been demonstrated to play a pivotal role in synthesis and metabolism of amyloid beta (Aβ) [3], a protein identified as the major component of the extracellular plaques in the central nervous system (CNS) that constitute the pathological hallmarks of Alzheimer’s disease (AD) [4]. In particular, the lipid-binding region of ApoE directly interacts with Aβ and promotes the Aβ transport out of the brain, thus enhancing peptide clearance [2]. Interestingly, ApoE ε4 shows the lowest affinity to Aβ, resulting in a significant reduction of Aβ clearance processes and the accumulation of Aβ toxic oligomers [3]. Concomitantly, ApoE ε4 alters the permeability of the blood–brain barrier (BBB), reducing the control of the passage of molecules, as Aβ peptide, from the brain to blood and vice versa [5]. The ε4 allele is the strongest polymorphism related to both early- and late-onset AD. Indeed, the ε4 homozygotes (ε4/ε4) are more predisposed to develop AD compared to ε4 heterozygotes (ε4/ε3 or ε4/ε2) and non-ε4 carriers (ε3/ε3, ε2/ε2, or ε3/ε2) subjects [2].

Additionally, the ApoE genotype is associated with oxidative stress, which is defined as an imbalance between an extreme generation of reactive oxygen species (ROS) and the lacking capability of the biological system to remove them [6]. The antioxidant defense mechanisms have been proven to be less efficient in the presence of ApoE ε4, as compared to other polymorphisms [6], causing the increase of ROS production, cellular damage, and alteration of proteins’ production and folding [7]. In particular, elevated oxidative damage in ε4 homozygotes has been linked to increased levels of lipid peroxidation [7], causing an alteration in the composition of the eukaryotic cellular membrane and its properties. Of note, an alteration of phosphatidylcholine (PC) and phosphatidylethanolamine (PE) ratio has been directly implicated in AD [8], along with a reduction in membrane fluidity of central and peripheral cells [9], thus strengthening the disease’s link with oxidative stress.

Several studies have widely confirmed that regular and moderate physical activity can prevent or at least slowdown brain aging and neurodegeneration [10,11]. In particular, exercise has been shown to modulate oxidative stress and upregulate antioxidant systems, to promote neurogenesis and angiogenesis and to increase the degradation of toxic oligomers [12]. In this regard, a recent study has demonstrated the negative correlation between physical activity and the accumulation of neurodegeneration-related proteins in peripheral cells [13]. In this sense, erythrocytes have been suggested as a good model to study the biochemical alteration correlated to aging/neurodegeneration and oxidative stress [14,15]. Indeed, erythrocytes are particularly sensitive to oxidative damage, due to the high concentration of oxygen and hemoglobin [16], and to neurodegeneration-related proteins accumulation, including Aβ [13,15,17].

A huge amount of data has reported that the effects of physical exercise training on systemic circulation and cognitive function are dependent on the ApoE genotype [18,19]. Nevertheless, the effects of physical activity on erythrocytes’ well-being depending on ApoE genotype remain to be elucidated.

On this basis, the current study aimed to evaluate the Aβ levels, the oxidative status together with the plasma antioxidant capability (AOC), and the membrane’s composition of erythrocytes in a cohort of human healthy subjects. These volunteers were analyzed considering their ApoE genotype and the level of physical activity, to establish how physical exercise can modulate the well-being of these cells, in the presence of ApoE polymorphism.

## 2. Materials and Methods

### 2.1. Subjects Recruitment and ApoE Genotyping

Forty-two age- and sex-matched (Table 1) healthy subjects were recruited from the Sport Medicine Unit of the Department of Clinical and Experimental Medicine of the University of Pisa. The volunteers were classified based on ApoE genotype, identified by the technique of restriction fragment length polymorphism (RFLP). Blood was collected from each subject and, subsequently, genomic DNA was extracted from whole blood. The polymerase chain reaction (PCR) was performed using 1.5 pmol of each primer (forward 5′-TCG-GCCGCA-GGG-CGC-TGA-TGG-3′ and reverse 5′-CTCGCG-GGC-CCC-GGC-CTG-GTA-3′), 250 μmol/L dNTPs, GC-rich (10% of the final volume), 2 units of Taq DNA polymerase (Applied Biosystems Inc., Branchburg, NJ), 10 ng/μL of genomic DNA, 25 mM MgCl_2_, and buffer 10×. Reactions were executed in a thermal cycler (PerkinElmer) for one cycle at 94 °C for 6 min, 30 cycles at 94 °C for 40 s, 67 °C for 30 s, 72 °C for 45 s, and a final extension at 72 °C for 5 min. Following a digestion with 3 U of HhaI restriction enzyme, the amplified fragments were separated exploiting agarose (5%) gel electrophoresis. The restriction patterns were visualized by ethidium bromide staining and UV light. The genotypes of subjects were defined by an ABI PRISM310 Automated Sequencer (Applied Biosystems, Forster City, CA, USA). In this way, the subjects were classified in ApoE ε4 carriers (twelve, seven female and five male, mean age 39 years, range 23–70) and ApoE non-ε4 carriers (twenty-six, fourteen female and twelve male, mean age 40 years, range 20–65). The minor amount of ApoE ε4 carriers is due to prevalence in human race to a lesser extent of this genotype compared to other polymorphisms of ApoE (ε2 or ε3), and, consequently, we were not able to predict the amount of ApoE ε4 carriers and non-ε4 carriers in the cohort of volunteers [2].

### 2.2. Study Population and Evaluation of the Physical Activity Level

For this study, Italian healthy volunteers with an upper-middle socio-economic status were recruited. All subjects showed no cardiovascular disease or other major medical disorders, as assessed by clinical history, physical examination, basal and stress electrocardiography, blood pressure, blood chemistry, hematology, and urine analysis, with a maximal graded cycle ergometry test performed by a cardiologist blinded to the other data [13,20]. Major inclusion criteria were as follows: Body mass index lower than 30 kg/m^2^, diastolic arterial blood pressure lower than 90 mmHg, systolic arterial blood pressure lower than 140 mmHg, total plasma cholesterol ranging from 120 to 220 mg/mL, high-density lipoproteins (HDL) cholesterol from 26 to 75 mg/mL, and plasma triglycerides from 30 to 150 mg/mL. Subjects were excluded if they were a smoker or received drug/nutraceutical treatments within 2 months before the beginning of the study [13].

The volunteers were divided into non-active and active based on the habits questionnaire. The subjects performing less than 150 min per week of physical activity were classified as non-active, according to the World Health Organization (WHO) [21]. Furthermore, the intensity level of physical activity was evaluated by the use of the Borg fating of perceived exertion (RPE) scale [22]. The scale ranges from 6 to 20, with 6 corresponding to no exertion at all, 7.5 to extremely light, 9 to very light, 11 to light, 13 to somewhat hard, 15 to hard, 17 to very hard, 19 to extremely hard, and 20 to maximal exertion.

The blood was collected from each subject at least 48 h after the last exercise bout. This study was approved by the Ethics Committee of the Great North West Area of Tuscany (271/2014 to F.F.) and it was carried out by the Declaration of Helsinki. All participants in the study were apprised: They received the informed consent and each one signed it giving their acceptance [13].

### 2.3. Blood Collection

The whole blood was sampled from each volunteer and it was conserved in an EDTA tube as anticoagulant. Centrifugation at 200× *g* at 4 °C for 10 min was required to separate erythrocytes from plasma. The plasma supernatant was isolated and frozen at −20 °C until use. The erythrocyte pellet was suspended in 3 mL of PBS, subjected to centrifugation at 1000× *g* for 10 min, and washed for three times with PBS. Erythrocytes were centrifuged at 1500× *g* for 10 min and frozen at −20 °C until use. The levels of AOC and lipid peroxidation were measured in plasma. The levels of Aβ, PC, PE, and the degree of membrane fluidity were analyzed in erythrocytes.

### 2.4. Quantification of Amyloid Beta (Aβ) in Erythrocytes

The levels of Aβ in erythrocytes were quantified by enzyme-linked immunosorbent assay (ELISA), as described [13]. The plate was coated with a 60 μL-well of a specific rabbit polyclonal antibody to Aβ (sc-9129, Santa Cruz Biotechnology), diluted 1:100 in poli-L-ornithine, and maintained overnight at 4 °C. Following extensive washing with PBS-T (PBS, containing 0.01% Tween 20), BSA 1% (200 μL-well) was added to block non-specific sites and incubated for 2 h at 37 °C. After washes with PBS-T, erythrocytes (0.05 mg-100 μL) were added to each well (100 μL-well) and incubated at 25 °C for 1 h. Following extensive washing, goat polyclonal antibody to Aβ (sc-5399, Santa Cruz Biotechnology) (75 μL-well), diluted 1:250, was employed for capturing and incubated for 1.5 h at 25 °C. After washing, for antigen detection, a donkey anti-goat-HRP antibody (Santa Cruz Biotechnology) (100 μL-well), diluted 1:2500 in PBS-BSA-Triton, was incubated at 37 °C for 1 h. The wells were then washed with PBS-T before the addition of 100 μL-well of 3,3′,5,5′-tetramethylbenzidine (TMB) (Thermo Scientific) (100 μL-well). The absorbance was evaluated at 450 nm after the addition of the Stop Solution (0.4 N HCl, 100 μL-well). All measurements were performed in duplicate to reduce inter-assay variability. The standard curve for ELISA assay was constructed using recombinant human Aβ solution at different concentrations diluted in PBS [13,17].

### 2.5. Evaluation of the Total Antioxidant Capability (AOC) in Plasma

The plasma AOC was evaluated by the total oxyradical scavenging capacity (TOSC) assay, a gas chromatographic assay that can determine oxyradical scavenging capacity of biological fluids [13,23]. Hydroxyl radicals were produced at 35 °C by the iron plus ascorbate-driven Fenton reaction (1.8 mM Fe^3+^, 3.6 mM EDTA, and 180 mM ascorbic acid in 100 mM PBS, pH 7.4). Reactions with 0.2 mM KMBA (alpha-keto gamma-methylthiobutyric acid) were carried out in 10 mL vials sealed with gas-tight Mininert valves (Supelco, Bellefonte, PA, USA) in a final volume of 1 mL. Ethylene production was measured by gas chromatographic analysis of 200 μL aliquots taken from the headspace of vials at timed intervals during the reaction (Hewlett-Packard gas chromatograph, HP 7820A Series, Andoven, M, equipped with a Supelco DB-1 capillary column and a flame ionization detector, FID). Total ethylene formation was quantified from the area under the kinetic curves that best define the experimental points obtained for control reactions and after the addition of plasma during the reaction [23,24]. TOSC values were measured from the equation TOSC = 100 − (SA/CA × 100), where SA is the area under the curve (AUC) for the sample and CA is the control reaction. A TOSC value of 100 is associated with a sample that suppresses the ethylene formation, while a pro-oxidant sample shows a negative TOSC value. A TOSC value of 0 corresponds to a sample with no scavenging capacity [25]. Each experiment was executed in duplicate to account for the intrinsic variability of the method. The results were expressed in TOSC units [23,26].

### 2.6. Lipid Peroxidation Assay in Plasma

The oxidative degradation of lipids was evaluated in plasma by measuring the levels of malondialdehyde (MDA), an end product of lipid peroxidation, through a fluorometric assay (Lipid Peroxidation (MDA) Assay Kit Colorimetric/Fluorimetric, Abcam, Cambridge, MA, USA, #ab118970).

Plasma (20 μL), isolated from whole blood, as previously described, was combined with 500 μL of H_2_SO_4_ (42 mM) and gently mixed. Then, 125 μL of phosphotungstic acid (PTA) solution was added and vortexed. The solution was incubated at room temperature for 5 min. After incubation, the solution was centrifuged at 13,000× *g* for 3 min. The pellet was collected and suspended using 100 μL of double-distilled H_2_O with 2 μL of butylated hydroxytoluene (BHT). Then, double-distilled H_2_O was added to reach a total volume of 200 μL. Following, 600 μL of 2-thiobarbituric acid (TBA) was added and the solution was incubated at 95 °C for 1 h. After incubation, cooling at room temperature in ice bath for 10 min was performed. The amount of MDA-TBA adduct was quantified by the relative fluorescence unit (RFU) at Ex/Em = 532/553 nm (EnSight Multimode Plate Reade, PerkinElmer, Waltham, MA, USA). The concentration of MDA (μM, i.e., nmol/mL) in the sample was calculated building a calibration curve.

### 2.7. Phosphatidylcholine (PC) Assay in Erythrocytes

The quantification of PC content in erythrocytes was performed using an enzyme-coupled reaction, which was able to hydrolyze PC and to release choline, which consequently oxidized the OxiRed probe, resulting in the development of fluorescence (Phosphatidylcholine Assay Kit Colorimetric/Fluorimetric, Abcam, #ab83377).

Erythrocytes (1 μL) were diluted in PC assay buffer and, subsequently, PC enzyme, PC developer, and the OxiRed probe were added. After incubation at room temperature for 30 min, the fluorescence was read (Ex/Em = 535/587 nm, EnSight Multimode Plate Reade, PerkinElmer). The quantity of PC (μM) in the sample was calculated by a standard curve built with different concentrations of PC standard.

### 2.8. Phosphatidylethanolamine (PE) Assay in Erythrocytes

The measurement of PE content in erythrocytes was carried out using a PE converter hydrolyses, that led to an intermediate, which converted a colorless probe to a fluorescent product through an enzymatic reaction (Phosphatidylethanolamine Assay Kit, Fluorimetric, BioVision, Milpitas, CA, USA, #K499-100). Erythrocytes (20 μL) were homogenized in a solution containing 5% Triton X-100 in double-distilled H_2_O. The samples were heated to 80 °C in a water bath for 10 min, cooled down, and then heated at the same temperature for the same time. The PE assay buffer and PE converter were added to samples and incubated at 45 °C for 1 h. Following, the PE developer and probe were added and the fluorescence was read (Ex/Em = 535/587 nm, EnSight Multimode Plate Reade, PerkinElmer). The amount of PE (μM) in the sample was calculated by a standard curve built with different concentrations of PE standard.

### 2.9. Membrane Fluidity Assay in Erythrocytes

The membrane fluidity of erythrocytes was measured using the lipophilic pyrene probe, a lipid analogue probe that underwent excimer formation upon spatial interaction with the cellular membrane (Membrane Fluidity Kit, Abcam, #ab189819).

This assay was performed using erythrocytes isolated from the whole blood and employed immediately, thus avoiding the freezing of the sample. Erythrocytes (1 mg of total proteins, measured by Bradford assay) were centrifuged at 1500× *g* for 10 min. The labelling solution (125 μL) was combined with the pellet and incubated at 25 °C for 20 min under continuous agitation. After a quick centrifugation, the pellet was washed with PBS and, after resuspension, centrifuged again. The perfusion buffer (50 μL) was added to the pellet and the fluorescence was read. By quantifying the ratio of excimer (Ex/Em = 350/470 nm) to monomer (Ex/Em = 350/370 nm) fluorescence (EnSight Multimode Plate Reade, PerkinElmer), quantitative monitoring of the membrane fluidity was realized. The data were expressed as the ratio between pyrene excimer and monomer (ratio Ie/Im).

### 2.10. Statistical Analysis

The data are presented as the mean value ± S.D. (standard deviation). The population included in this study presented a normal distribution for age. Kolmogorov–Smirnov tests were applied to data meeting the assumption of a normality distribution. One-way analysis of variance (ANOVA) tests were used for data meeting the assumption of homogeneity of variance. Pearson correlation analysis and t tests were applied for data with distributions that met parametric assumptions. Chi-square tests (Pearson’s, Yates-adjusted or Fisher’s exact test according to sample size), Mann–Whitney U tests, and Spearman correlation analysis were used in situations where parametric assumptions were not met. Differences among groups were evaluated by One-way analysis of variance (ANOVA). When only two groups were present, an unpaired t-test was used. Correlation between variables was determined by linear regression analysis, while interactions between variables were calculated by correlation and multiple regression analyses. *P*-values < 0.05 were deemed significantly different. All statistical procedures were performed by commercial software (GraphPad Prism, version 7.0; GraphPad Software Inc., San Diego, CA, USA).

Correlation between variables was determined by simple linear regression analysis, while covariate analysis was performed by the partial correlation matrix. Finally, a multiple linear regression analysis was performed to assess the effects of ApoE on the relationship between Aβ concentrations and physical activity level. All statistical procedures were performed using the StatView program (Abacus Concepts, Inc., SAS Institute, Cary, NC, USA) [17,24].

## 3. Results

### 3.1. Descriptive Statistics

The whole cohort of healthy subjects (*n* = 42, Table 1) was divided into ApoE ε4 carriers (mean age 39.33 ± 14.25) and ApoE non-ε4 carriers (mean age 39.92 ± 12.65). The subjects were further classified in non-active and active, based on the results of the Borg score (see Methods section).

The active and non-active subjects did not present significant differences in age, sex distribution, and body mass index (BMI). Not surprising, the level of physical activity in the active was higher than in non-active subjects (*P* < 0.0001).

Table 1 summaries the mean values of glucose, cholesterol, and triglycerides of the recruited subjects. Subjects carrying ε4 showed significantly lower levels of HDL (*P* = 0.0105) and higher levels of LDL (*P* = 0.0226). Nevertheless, total levels of cholesterol were comparable between ApoE ε4 carriers and non-ε4 carriers, although a trend in lower levels was noticed in the latter group (*P* = 0.0569). This trend reached a significance in the active subgroup (A ε4 carriers vs. A non-ε4 carriers *P* = 0.0190). Triglycerides (*P* = 0.0899) and glucose (*P* = 0.3107) did not significantly differ between ApoE ε4 carriers and non-carriers.

The additional biochemical parameters measured in this study are summarized in Table 2.

### 3.2. Antioxidant Capability (AOC) in Plasma

Oxidative stress has been suggested as a representative marker of aging [27]. Particularly, aging and the ApoE ε4 genotype have been related to a defective ability to respond to cellular stress [28].

**Figure 1:**
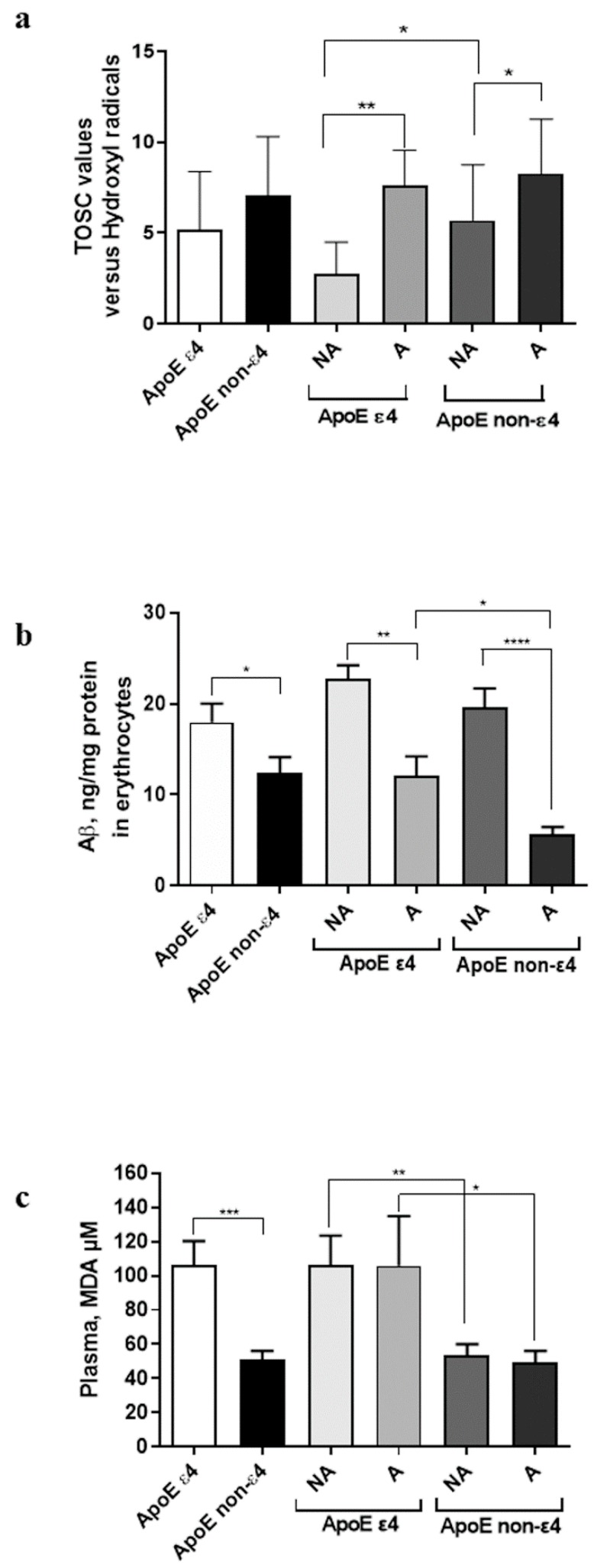
Plasma AOC levels, erythrocyte Aβ accumulation, and plasma lipid peroxidation amount in active and non-active subjects depending on ApoE genotype. (**a**) Plasma AOC depends on ApoE genotype and increases with physical activity. High TOSC values are associated with elevated antioxidant capacity. (**b**) Aβ accumulation in erythrocytes is influenced by ApoE genotype and decreases with physical activity. (**c**) Lipid peroxidation is higher in ApoE ε4 carriers and is not modulated by physical activity. The data are presented as the mean value ± S.D. and are representative of three independent experiments (*n* = 3). Difference among groups were assessed by One-way ANOVA. *P*-values were adjusted with Sidak’s multiple comparison test: * *P* < 0.05, ** *P* < 0.01, *** *P* < 0.001, **** *P* < 0.0001 between the indicated subgroups.

On this basis, the AOC in plasma was assessed using the TOSC assay (Figure 1a), in which higher levels of TOSC are associated to an improved AOC.

ApoE non-ε4 carriers showed higher levels of AOC than the ε4 carriers in the whole group (*P* = 0.0290), and in particular among non-active subjects (non-active ε4 carriers vs. non-active non-ε4 carriers, *P* = 0.0361). These data confirm that the ApoE ε4 polymorphism can represent a causative factor of the decrease of the plasma AOC.

Moreover, active subjects displayed increased AOC compared to non-active subjects, even if the statistical significance was reached in the ε4 carriers only (carriers: *P* = 0.0036; non-ε4 carriers: *P* = 0.1514). The results confirm that physical activity can modulate plasma AOC, even in the presence of ApoE ε4 polymorphism.

### 3.3. Levels of Amyloid Beta (Aβ) in Erythrocytes

Together with the ApoE ε4 carriage, aging is the main trigger event that causes Aβ accumulation and aggregation [29]. In this sense, Aβ has been recently demonstrated to accumulate in erythrocytes, in both humans and animal models of AD [30,31,32].

Herein, the levels of Aβ in erythrocytes were evaluated by a specific immunoenzymatic assay (Figure 1b). The subjects presenting the ApoE ε4 polymorphism displayed a higher Aβ concentration in erythrocytes compared to the non-ε4 carriers in the whole population (*P* = 0.0212), and in the active (active ε4 carriers vs. active non-ε4 carriers *P* = 0.0101), confirming that the ε4 polymorphism is associated with Aβ accumulation in erythrocytes.

Moreover, active subjects showed reduced levels of the amylogenic protein compared to non-active subjects in both ε4 carriers (*P* = 0.0090) and non-ε4 carriers (*P* < 0.0001), thus suggesting that regular exercise can decrease Aβ accumulation in erythrocytes, independently from ApoE genotype. Globally, these results showed that Aβ levels are influenced by both ApoE polymorphism and physical activity.

### 3.4. Lipid Peroxidation in Plasma

To evaluate the oxidative degradation of lipids due to oxidative stress, MDA concentration in plasma was quantified (Figure 1c). Indeed, MDA is one of the most important intermediate/end-product of lipid peroxidation [7], which is increased in aging [33,34].

Increased plasma lipid peroxidation was shown by ApoE ε4 carriers compared to non-ε4 carriers in the whole population (*P* = 0.0001), as well as in the non-active group (non-active ε4 carriers vs. non-active non-ε4 carriers, *P* = 0.0034) and in the active group (active ε4 carriers vs. active non-ε4 carriers, *P* = 0.0319). These data suggest that the ε4 polymorphism could be a possible causative factor of the increase of lipid peroxidation, independently from the physical activity level performed by the subjects. Consistent with this hypothesis, non-active and active subjects presented comparable levels of MDA (ε4 carriers: *P* = 0.9333, non-ε4 carriers: *P* = 0.5446).

Overall, the data revealed that lipid peroxidation depends on ApoE ε4 polymorphism and it is not influenced by the levels of physical activity.

### 3.5. Phosphatidylcholine (PC) and Phosphatidylethanolamine (PE) Amount in Erythrocytes

The oxidative stress occurs constantly on erythrocytes in healthy subjects, and it significantly increases with age, particularly changing the composition of membrane phospholipids, including PC and PE [35].

**Figure 2:**
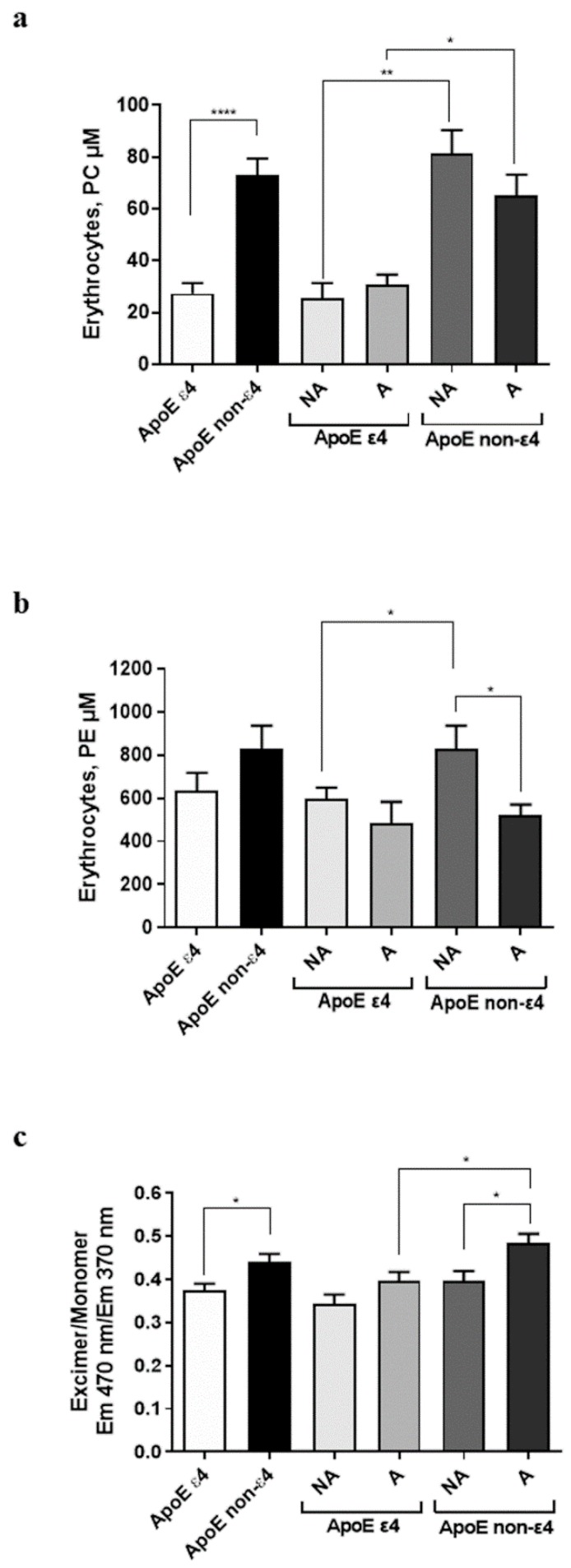
Membrane composition and membrane fluidity in active and non-active subjects depending on ApoE genotype. (**a**,**b**) The amount of PC and PE is differently affected by ApoE polymorphism and physical activity. The results are expressed in terms of concentration of PC (μM) and PE (μM). (**c**) Membrane fluidity of erythrocytes is negatively affected by ApoE ε4 polymorphism, but positively modulated by physical exercise. By determining the ratio of the fluorescence of excimer (Ex/Em = 350/470 nm) to the fluorescence of monomer (Ex/Em = 350/370 nm), quantitative monitoring of membrane fluidity is achieved. Elevated levels of the excimer to monomer fluorescence ratio (Ie/Im) reveal a higher membrane fluidity. The data are presented as the mean value ± S.D. and are representative of three independent experiments (*n* = 3). Differences among groups were assessed by one-way ANOVA. *P*-values were adjusted with Sidak’s multiple comparison test: * *P* < 0.05, ** *P* < 0.01, **** *P* < 0.0001 between the indicated subgroups.

Thus, the amount of PC in erythrocytes was assessed as representative phospholipid of the cellular membrane (Figure 2a). ApoE non-ε4 carriers showed significantly higher PC levels in erythrocytes with respect to ε4 carriers, in the whole population (*P* < 0.0001), as well as in active (active ε4 carriers vs. active non-ε4 carriers, *P* = 0.0280) and in non-active subjects (non-active ε4 carriers vs. non-active non-ε4 carriers, *P* = 0.0027). These data demonstrate that the presence of ε4 polymorphism can reduce PC content in erythrocytes, independently from the physical activity level of the subjects.

Consistently, comparable levels of erythrocyte PC were found between active and non-active subjects (ε4 group: *P* = 0.3929; non-ε4 group: *P* = 0.2475). Globally, these data demonstrate that PC levels are dependent on ApoE polymorphism, but not on physical activity.

Moreover, the contents of PE in erythrocytes was measured in the same subjects, as depicted in Figure 2b. The PE amount in erythrocytes was comparable between ε4 carriers and non-ε4 carriers (*P* = 0.1717), even if a trend of increase was shown by the latter group. This statistical significance was reached in the non-active subgroup, in which ε4 carriers showed a significant minor amount of PE in erythrocytes compared to the non-ε4 carriers (*P* = 0.0473). Moreover, non-active subjects displayed an increased amount of PE in erythrocytes compared to the respective active in the non-ε4 carriers (*P* = 0.0232), but not in the ε4 group (*P* = 0.6158). These data suggest that physical activity can affect PE content in the absence of ε4 polymorphism.

### 3.6. Membrane Fluidity of Erythrocytes

The lipid peroxidation may represent a pivotal factor in the alteration of the membrane fluidity, which occurs during the aging process [36].

In this respect, the level of membrane fluidity of erythrocytes (Figure 2c) was evaluated to establish the dynamic properties of cell membranes, by quantifying the ratio of the excimer to monomer: a gain of the ratio corresponds to increased membrane fluidity.

ApoE non-ε4 carriers presented a higher membrane fluidity than carriers in the whole population (*P* = 0.0322) and among active (active ε4 carriers vs. active non-ε4 carriers, *P* = 0.0421). These data suggest that the presence of the ε4 polymorphism affects negatively membrane fluidity.

Furthermore, active displayed increased levels of membrane fluidity compared to non-active subjects in the non-ε4 carriers (*P* = 0.0421), thus suggesting that physical exercise can enhance erythrocytes membrane fluidity in the absence of the ApoE polymorphism.

### 3.7. Correlation among Triglycerides, Cholesterol, and Glucose Levels

Simple regression analyses were performed to verify the putative correlation between cholesterol, triglycerides, or glucose levels and the plasma AOC or the erythrocytes well-being parameters.

Interestingly, plasma MDA concentration was inversely related to the HDL levels (*P* = 0.0162, R^2^ = 0.203).

The plasma AOC versus hydroxyl radicals was inversely related to the total cholesterol (*P* = 0.0392, R^2^ = 0.148) and LDL concentration (*P* = 0.0487, R^2^ = 0.141). In contrast, Aβ concentration in erythrocytes was directly correlated with LDL concentrations (*P* = 0.0394, R^2^ = 0.153).

### 3.8. Correlation between Plasma and Erythrocytes Well-Being Parameters

**Figure 3:**
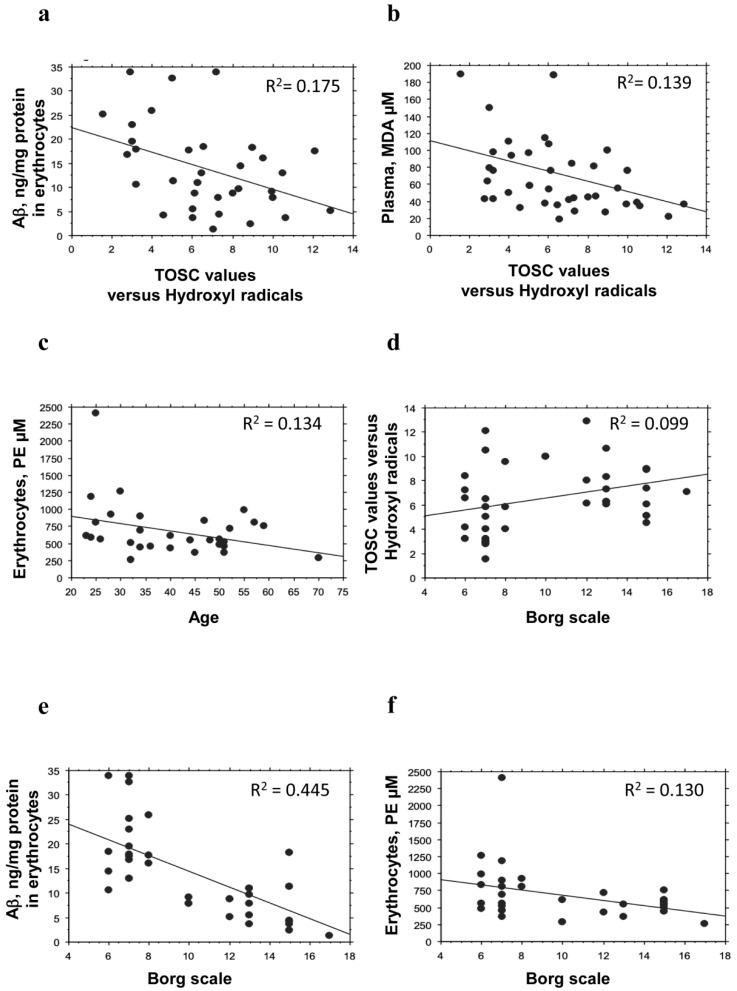
Correlation between plasma and erythrocytes well-being parameters. Correlation analysis between erythrocytes Aβ (**a**) or lipid peroxidation (**b**) and plasma AOC. (**c**) Correlation analysis between PE and age. Correlation analysis between plasma AOC (**d**), Aβ (**e**), or PE concentration (**f**) and Borg scale. Correlation between variables was determined by simple linear regression analysis, using the StatView program (Abacus Concepts, Inc., SAS Institute, Cary, NC). *P* and R^2^ values obtained for each correlation are reported in the respective panel.

The plasma AOC showed significant inverse correlation with the erythrocyte’s levels of Aβ (Figure 3a, *P* = 0.0089, R^2^= 0.175) and lipid peroxidation (Figure 3b, *P* = 0.0150, R^2^= 0.139). These data demonstrate that negative alterations of erythrocytes’ well-being are possibly linked to the plasma antioxidant ability.

In contrast, TOSC values were not related to the other examined parameters (PE: *P* = 0.1352; PC: *P* = 0.2771; membrane fluidity: *P* = 0.2698).

### 3.9. Correlation of Plasma and Erythrocytes Well-Being Parameters with Age and the Level of Physical Activity

The amount of PE in erythrocytes was inversely related to age (Figure 3c, *P* = 0.0359, R^2^ = 0.134). No significant correlation with age was evidenced for the other parameters.

The physical activity level showed a direct correlation with the plasma AOC (Figure 3d, *P* = 0.0428, R^2^ = 0.099), as previously obtained in a different cohort of subjects [17].

A significant inverse correlation was observed between the level of physical exercise and Aβ (Figure 3e, *P* < 0.0001, R^2^ = 0.445) or PE (Figure 3f, *P* = 0.0394, R^2^ = 0.130) accumulation in erythrocytes. These data confirm that the protein levels in these blood cells may be influenced by exercise.

The physical activity level was not significantly related to the other examined parameters (lipid peroxidation: *P* = 0.8171; PC concentration: *P* = 0.3885; membrane fluidity: *P* = 0.02558).

### 3.10. Covariate and Multiple Regression Analyses

Following covariate analysis for ApoE polymorphism, the correlation between the Borg score and the aforementioned variables was lost for statistical significance (plasma AOC: *P* = 0.5453; Aβ: *P* = 0.0530; PE: 0.1918).

These results confirm that the ApoE polymorphism influences the effect of the physical activity level on the plasma and erythrocytes’ well-being.

**Figure 4:**
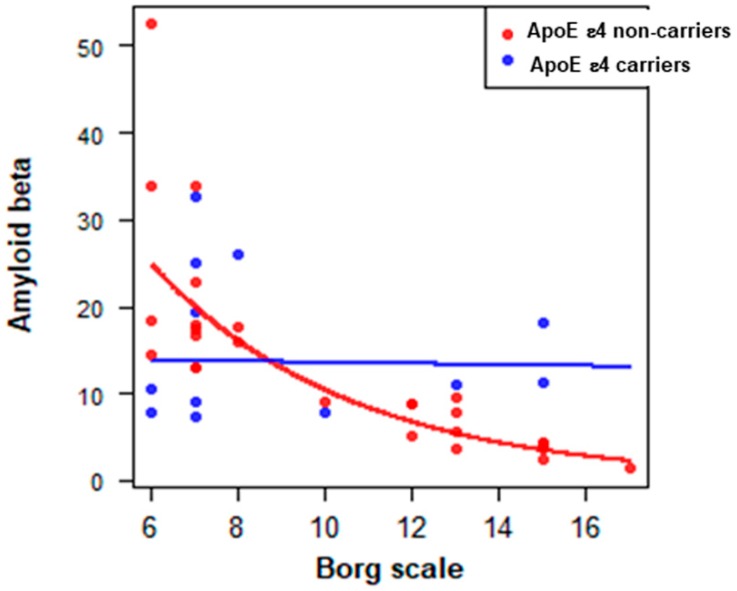
Multiple linear regression analysis in ε4-non carriers and ε4 carriers. The analysis was used to assess the relationship between erythrocytes Aβ concentration and physical activity level in ε4 carriers and non-ε4 carriers.

Finally, a multiple linear regression analysis was performed to assess the effects of ApoE on the relationship between Aβ concentrations and physical activity level. This analysis confirmed that the decrease in erythrocytes Aβ levels in non-ε4 carriers was strictly dependent on the level of physical activity (Figure 4). In contrast, the decrease in Aβ concentration was completely independent of the level of physical activity in non-ε4 carriers (Figure 4).

## 4. Discussion

In the current study, the influence of ApoE polymorphism and physical activity on the Aβ contents, oxidative status, and membrane’s composition of erythrocytes was evaluated. The main results of the paper are as follows: (i) Plasma AOC, erythrocytes PC content, and membrane fluidity were significantly decreased in ApoE ε4 subjects; (ii) the erythrocytes concentration of Aβ and plasma lipid peroxidation increased in ε4 carriers when compared to non-ε4 carriers; (iii) regular physical exercise was associated to increased plasma AOC and membrane fluidity, as well as to a reduced Aβ amount and a minor PE content. Taken together, these data highlight the influence of ApoE genotype on blood parameters of well-being and confirm the positive impact of moderate physical exercise.

The presence of the ε4 allele has been widely related to higher levels of plasma cholesterol, especially LDL-cholesterol [37,38,39], and non-fasting serum triglyceride values [40,41]. In our study, and according to literature data, subjects carrying ε4 presented significantly lower levels of HDL and higher levels of LDL. In contrast, triglycerides showed a trend of increase in ApoE ε4 individuals when compared to non-carriers, without reaching statistical significance. This discrepancy may be ascribed to the limited cohort of subjects carrying the ApoE polymorphism.

Besides lipid metabolism, ApoE ε4 polymorphism has been related to an alteration of Aβ levels, oxidative status, and membrane’s composition, even before the onset of AD [8,9]. In this sense, nowadays, the study of the cellular and molecular pathways which lead to AD [42], and the investigation of AD prognostic markers, are wide themes of research [43]. However, it has been proven that regular and moderate physical activity can reduce the accumulation of toxic oligomers, modulate the levels of oxidative stress, and promote neurogenesis and angiogenesis [12].

In the present paper, erythrocytes were chosen as a good peripheral model to study biochemical alteration of aging [44]. Indeed, it has been supposed that, in blood, Aβ that accumulates with aging binds the membrane of erythrocytes, which are particularly susceptible to oxidative stress, modifying their morphology and damaging their functions [45]. Our study confirmed that the increased amount of Aβ occurs particularly in ApoE ε4 carriers compared to non-ε4 carriers, as previously demonstrated [2]. These results are consistent with literature data demonstrating that ApoE ε4 is a determinative factor for Aβ deposition [46], even in peripheral fluids [47]. In our study, Aβ accumulation in erythrocytes directly correlated with LDL concentrations, thus highlighting the link between the ApoE polymorphism and amyloid metabolism.

Uniquely, our results highlighted that the levels of Aβ in erythrocytes are meaningfully regulated by physical activity: Active subjects showed significantly lower Aβ levels compared to non-active subjects in both ε4 carriers and non-ε4 carriers, and Aβ concentration was inversely related to the level of physical exercise in the whole group. Similarly, physical exercise has been shown to reduced cerebral [48] and plasma [49] Aβ.

Interestingly, multiple regression analysis confirmed that the decrease in erythrocytes Aβ levels in non-ε4 carriers was strictly dependent on the level of physical activity. In contrast, the decrease in Aβ concentration was completely independent of the level of physical activity in non-ε4 carriers. Similarly, the benefits of a physical activity-induced decrease in plasma Aβ amount have been shown to be received by non-ε4 carriers only [50]. Of note, the effect observed in non-ε4 carriers is related to moderate physical exercise, because extreme physical exercise has an opposite effect, even in the liver, due to damage of erythrocytes, which may cause a Fenton-type reaction as an effect of the release of free iron from erythrocytes [51].

Among the alterations caused by the accumulation of Aβ in erythrocytes, increased oxidative stress has been surely emerging [15]. In particular, oxidative stress has been demonstrated to be elevated in ApoE ε4 carriers and to increase physiologically with aging [27].

According to these findings, our results showed an inverse correlation between plasma AOC towards hydroxyl radicals and the Aβ erythrocytes levels, and a significant decrease in ApoE ε4 carriers compared to non-ε4 carriers. Moreover, physical activity was confirmed to modulate the antioxidant capability, independently from ApoE polymorphism. Indeed, active subjects showed higher levels of plasma AOC compared to non-active subjects, and plasma AOC showed a significant direct correlation with the level of physical activity, as previously reported in human subjects [13,17,52].

Oxidative stress has been linked to elevated lipid peroxidation in ApoE ε4 carriers [7] and, consequently, in AD patients [53] and generally within aging [33]. In our study, plasma lipid peroxidation was significantly higher in ApoE ε4 carriers when compared to non-ε4 carriers, and showed an inverse correlation with the plasma AOC. Consistent with these findings, this ApoE isoform has been proven to decrease the peroxidation index of human neuroblastoma cells [54]. Furthermore, non-active and active subjects presented comparable levels of lipid peroxidation. In contrast, hepatic MDA levels have been proven to decrease in all age groups following chronic physical activity in mice [55]. These discrepancies may be explained considering the major role of the hepatic tissue in the antioxidant responses.

Oxidative stress and lipid peroxidation have been related to lipid profile too. For instance, a decrease in the cholesterol LDL/HDL ratio has been associated with minor lipid peroxidation that has been noticed following a lettuce diet [56]. Similarly, an improvement in the antioxidant status, following lycopene supplementation, has been proven to optimize the plasma lipid profile, with reduced levels of plasma total cholesterol and LDL [57]. Consistently, our data showed an inverse correlation between plasma AOC and total cholesterol/LDL concentration, and plasma MDA concentration was inversely related to the HDL levels. Globally, these data confirm the link between plasma oxidative status, lipid peroxidation, and blood lipid profile.

The oxidative stress cause alterations in the membrane’s composition and properties, especially during aging [35]. A reduction of PC/PE (i.e., decreased levels of PC and increased levels of PE) leads to alterations in the metabolic profile, which characterize some diseases, such as AD [58]. Our results showed that the presence of ε4 polymorphism reduced the PC content in erythrocytes. Consistently, ApoE ε4 has been associated with phospholipid dysregulation, contributing to the development of tau hyper-phosphorylation in the brain [59]. The observed effects on erythrocytes PC occurred independently from the physical activity level of the subjects, as previously observed in erythrocytes membranes [60].

Differently from PC content, the amount of PE in erythrocytes decreased in ε4 carriers, particularly in the presence of a non-active lifestyle. On the whole, non-active ApoE ε4 carriers showed a high ratio PC:PE, suggesting their greatest attitude to membranes alteration, as compared to the other groups.

The alteration of membrane fluidity is a consequence of high oxidative stress and modification in membrane phospholipids amount, which occurs in aging [36] and characterizes some diseases, including AD [9]. Herein, ApoE ε4 carriers displayed lower levels of membrane fluidity than non-ε4 subjects. Consistent with the aforementioned data on membrane phospholipids, erythrocyte membrane fluidity has been proven to decrease following a decrease in phospholipid content [61].

In contrast, subjects undergoing regular physical exercise showed improved dynamic properties of the cellular membranes. These data are consistent with the literature, revealing that physical exercise increased erythrocyte membrane fluidity [62].

**Figure 5:**
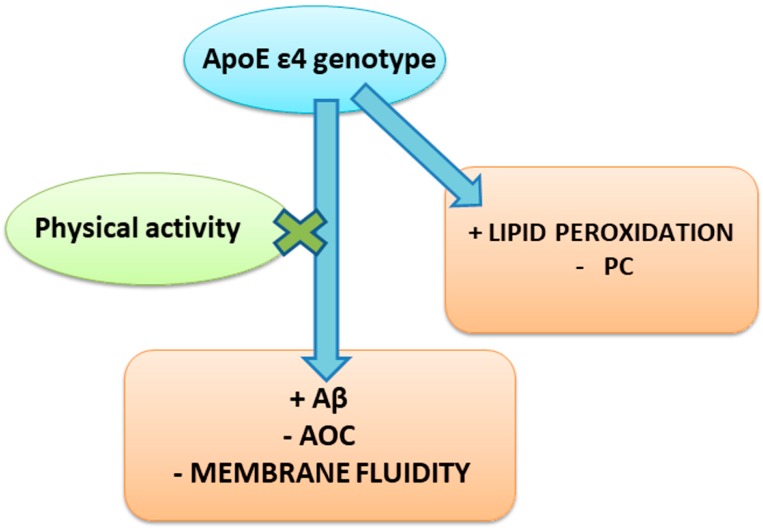
Modulation of the Aβ contents, oxidative status, and membrane’s composition of erythrocytes by ApoE polymorphism and physical activity. The presence of the ApoE ε4 genotype enhances Aβ levels and lipid peroxidation, and decreases plasma AOC, membrane fluidity, and PC amount in erythrocytes. Regular physical activity counteracts the negative effects exhibited by the ε4 genotype on Aβ levels, plasma AOC, and membrane fluidity.

Taken together, our results (Figure 5) suggest erythrocytes as an adequate model to monitor biochemical alterations and to study how their well-being is related to the ApoE polymorphism and physical activity. In particular, we demonstrated that regular PA reduced Aβ accumulation and lipid peroxidation shown by ε4 carriers, and counteracted the reduction of membrane fluidity and plasma AOC shown by the same subjects (Figure 5).

This is a pilot study that stresses the positive effects of physical activity on the antioxidant capability and, generally, on the erythrocyte’s well-being, particularly in those subjects presenting the ApoE ε4 genotype. However, our study presents some limitations. One of these regards the unfeasibility of measuring the turnover of erythrocytes. Conflicting data about the alteration of erythropoiesis are available in the literature [63]. Nevertheless, the influence of physical exercise on the aging and turnover of erythrocytes could be taken into account in a future study. The other restriction concerns the modest number of subjects recruited in the study, which highlights the need to confirm our preliminary data.

## Figures and Tables

**Table 1:** Analysis of the total cohort of healthy subjects.

Groups	Number of Subjects (n)	Age (Years)	Physical Activity Level (Borg Scale)	Glucose (mg/mL)	Cholesterol (mg/mL)	HDL (mg/mL)	LDL (mg/mL)	Triglycerides (mg/mL)
ApoE ε4 carriers	16	39.3 ± 14.2	9.0 ± 3.4	89.2 ± 8.36	198 ± 19.7	53.1 ± 9.26	125 ± 21.7	113 ± 35.8
ApoE non-ε4 carriers	26	39.9 ± 12.6	10.2 ± 3.6	73.0 ± 26.6	168 ± 66.3	57.6 ± 23.2	98.5 ± 39.3	75.3 ± 30.9
NA ApoE ε4 carriers	8	38.2 ± 11.3	6.88 ± 0.64	84.2 ± 7.92	205 ± 14.2	55.2 ± 10.3	133 ± 11.7	106.2 ± 44.1
A ApoE ε4 carriers	8	41.5 ± 21.0	13.2 ± 2.36	95.5 ± 2.89	194 ± 22.3	51.7 ± 9.02	119 ± 25.4	117 ± 32.0
NA ApoE non-ε4 carriers	13	40.8 ± 13.8	6.65 ± 0.69	76.2 ± 21.6	198 ± 35.8	65.1 ± 10.0	118 ± 30.6	82.0 ± 23.4
A ApoE non-ε4 carriers	13	39.0 ± 11.9	13.5 ± 1.9	89.0 ± 8.69	174 ± 16	64.7 ± 13.4	92.4 ± 17.3	105.9 ± 35.7

The subjects are classified in ApoE ε4 carriers and ApoE non-ε4 carriers; each of them is further classified in non-active (NA) and active (A) on the base of physical activity level (see Methods section). For each group, the number of recruited subjects (N) and the age (years) are indicated. Glucose, cholesterol, high-density lipoproteins (HDL) cholesterol, low-density lipoproteins (LDL) cholesterol and triglycerides are reported in mg/mL. Values are expressed as mean ± SD.

**Table 2:** Values of erythrocyte and plasma parameters analyzed in the study.

Groups	Aβ (ng/mg)	AOC (TOSC Values)	Lipid Peroxidation (MDA, μM, i.e., nmol/mL)	PC (μM)	PE (μM)	Membrane Fluidity (E/M)
ApoE ε4 carriers	18.0 ± 8.65	5.19 ± 3.18	106 ± 49.0	27.4 ± 11.0	635 ±2 61	0.37 ± 0.06
ApoE non-ε4 carriers	12.4 ± 8.82	7.06 ± 3.24	51.4 ± 23.8	73.2 ± 27.8	832 ± 333	0.44 ± 0.08
NA ApoE ε4 carriers	22.7 ± 8.24	2.76 ± 1.73	106 ± 48.3	25.4 ± 13.2	596 ± 223	0.34 ± 0.05
A ApoE ε4 carriers	12.1 ± 4.37	7.63 ± 1.91	106 ± 57.9	30.8 ± 6.7	485 ± 170	0.40 ± 0.06
NA ApoE non-ε4 carriers	19.6 ± 7.19	5.69 ± 3.06	53.4 ± 24.2	81.4 ± 28.4	832 ± 333	0.40 ± 0.07
A ApoE non-ε4 carriers	5.70 ± 2.77	8.28 ± 2.97	49.5 ± 24.2	65.0 ± 25.9	523 ± 152	0.48 ± 0.06

The values of erythrocyte Aβ (ng/mg protein), PC (μM), PE (μM), membrane fluidity (expressed as ratio of Excimer to Monomer), plasma AOC (expressed in terms of TOSC values) and lipid peroxidation (MDA concentration, μM) are indicated in the table for each analyzed group. Values are expressed as mean ± SD.

## References

[1] Giau V.V., Bagyinszky E., An S.S., Kim S.Y. (2015). Role of apolipoprotein E in neurodegenerative diseases. Neuropsychiatr. Dis. Treat..

[2] Liu C.C., Liu C.C., Kanekiyo T., Xu H., Bu G. (2013). Apolipoprotein E and Alzheimer disease: risk, mechanisms and therapy. Nat. Rev. Neurol..

[3] Mahley R.W., Rall S.C. (2000). Apolipoprotein E: Far more than a lipid transport protein. Annu. Rev. Genom. Hum. Genet..

[4] Serrano-Pozo A., Frosch M.P., Masliah E., Hyman B.T. (2011). Neuropathological alterations in Alzheimer disease. Cold Spring Harb. Perspect. Med..

[5] Bell R.D., Winkler E.A., Singh I., Sagare A.P., Deane R., Wu Z., Holtzman D.M., Betsholtz C., Armulik A., Sallstrom J. (2012). Apolipoprotein E controls cerebrovascular integrity via cyclophilin A. Nature.

[6] Ross J.M., Olson L., Coppotelli G. (2015). Mitochondrial and ubiquitin proteasome system dysfunction in ageing and disease: two sides of the same coin?. Int. J. Mol. Sci..

[7] Ramassamy C., Averill D., Beffert U., Bastianetto S., Theroux L., Lussier-Cacan S., Cohn J.S., Christen Y., Davignon J., Quirion R. (1999). Oxidative damage and protection by antioxidants in the frontal cortex of Alzheimer’s disease is related to the apolipoprotein E genotype. Free Radic. Biol. Med..

[8] Calzada E., Onguka O., Claypool S.M. (2016). Phosphatidylethanolamine metabolism in health and disease. Int. Rev. Cell Mol. Biol..

[9] Ortiz G.G., Pacheco-Moisés F.P., Flores-Alvarado L.J., Macías-Islas M.A., Velázquez-Brizuela I.E., Ramírez-Anguiano A.C., Tórres-Sánchez E.D., Moráles-Sánchez E.W., Cruz-Ramos J.A., Ortiz-Velázquez G.E. (2013). Alzheimer Disease and Metabolism: Role of Cholesterol and Membrane Fluidity.

[10] Morris J.K., Vidoni E.D., Johnson D.K., van Sciver A., Mahnken J.D., Honea R.A., Wilkins H.M., Brooks W.M., Billinger S.A., Swerdlow R.H. (2017). Aerobic exercise for Alzheimer’s disease: A randomized controlled pilot trial. PLoS ONE.

[11] Paillard T., Rolland Y., de Souto Barreto P. (2015). Protective effects of physical exercise in Alzheimer’s Disease and Parkinson’s Disease: a narrative review. J. Clin. Neurol..

[12] Radak Z., Suzuki K., Higuchi M., Balogh L., Boldogh I., Koltai E. (2016). Physical exercise, reactive oxygen species and neuroprotection. Free Radic. Biol. Med..

[13] Daniele S., Frosini D., Pietrobono D., Petrozzi L., Lo Gerfo A., Baldacci F., Fusi J., Giacomelli C., Siciliano G., Trincavelli M.L. (2018). α-synuclein heterocomplexes with β-amyloid are increased in red blood cells of Parkinson’s Disease patients and correlate with disease severity. Front. Mol. Neurosci..

[14] Singh S. (2015). Antioxidants as a preventive therapeutic option for age related neurodegenerative disease. Targets Neurol. Dis..

[15] Kiko T., Nakagawa K., Satoh A., Tsuduki T., Furukawa K., Arai H., Miyazawa T. (2012). Amyloid β levels in human red blood cells. PLoS ONE.

[16] Karsten E., Breen E., Herbert B.R. (2018). Red blood cells are dynamic reservoirs of cytokines. Sci. Rep..

[17] Daniele S., Pietrobono D., Fusi J., Iofrida C., Chico L., Petrozzi L., Lo Gerfo A., Baldacci F., Galetta F., Siciliano G. (2018). α-synuclein aggregates with β-amyloid or tau in human red blood cells: correlation with antioxidant capability and physical exercise in human healthy subjects. Mol. Neurobiol..

[18] Lee J.H., Hong S.M., Shin Y.A. (2018). Effects of exercise training on stroke risk factors, homocysteine concentration, and cognitive function according the APOE genotype in stroke patients. J. Exerc. Rehabil..

[19] Solomon A., Turunen H., Ngandu T., Peltonen M., Levälahti E., Helisalmi S., Antikainen R., Bäckman L., Hänninen T., Jula A. (2018). Effect of the Apolipoprotein E genotype on cognitive change during a multidomain lifestyle intervention: a subgroup analysis of a randomized clinical trial. JAMA Neurol..

[20] Whaley M.H., Brubaker P.H., Otto R.M., Armstrong L.E. (2006). Medicine ACoS Guidelines for Exercise Testing and Prescription.

[21] Wicker P., Frick B. (2017). Intensity of physical activity and subjective well-being: An empirical analysis of the WHO recommendations. J. Public Health.

[22] Borg G.A. (1982). Psychophysical bases of perceived exertion. Med. Sci. Sports Exerc..

[23] Regoli F., Winston G.W. (1999). Quantification of total oxidant scavenging capacity of antioxidants for peroxynitrite, peroxyl radicals, and hydroxyl radicals. Toxicol. Appl. Pharm..

[24] Franzoni F., Ghiadoni L., Galetta F., Plantinga Y., Lubrano V., Huang Y., Salvetti G., Regoli F., Taddei S., Santoro G. (2005). Physical activity, plasma antioxidant capacity, and endothelium-dependent vasodilation in young and older men. Am. J. Hypertens..

[25] Bianchi S., Fusi J., Franzoni F., Giovannini L., Galetta F., Mannari C., Guidotti E., Tocchini L., Santoro G. (2016). Effects of recombinant human erythropoietin high mimicking abuse doses on oxidative stress processes in rats. Biomed. Pharm..

[26] Franzoni F., Colognato R., Galetta F., Laurenza I., Barsotti M., Di Stefano R., Bocchetti R., Regoli F., Carpi A., Balbarini A. (2006). An in vitro study on the free radical scavenging capacity of ergothioneine: Comparison with reduced glutathione, uric acid and trolox. Biomed. Pharm..

[27] Muralidharan N., Bhat T., Kumari S.N. (2017). A study on effect of ageing on the levels of total antioxidant and lipid peroxidation. Int. J. Contemp. Med. Res..

[28] Dose J., Huebbe P., Nebel A., Rimbach G. (2006). APOE genotype and stress response—A mini review. Lipids Health Dis..

[29] Lim Y.Y., Mormino E.C. (2017). APOE genotype and early β-amyloid accumulation in older adults without dementia. Neurology.

[30] Baldacci F., Daniele S., Piccarducci R., Giampietri L., Pietrobono D., Giorgi F.S., Nicoletti V., Frosini D., Libertini P., Lo Gerfo A. (2019). Potential diagnostic value of red blood cells α-synuclein heteroaggregates in Alzheimer’s Disease. Mol. Neurobiol..

[31] Piccarducci R., Pietrobono D., Pellegrini C., Daniele S., Fornai M., Antonioli L., Trincavelli M.L., Blandizzi C., Martini C. (2019). High Levels of β-Amyloid, Tau, and Phospho-Tau in Red Blood Cells as Biomarkers of Neuropathology in Senescence-Accelerated Mouse. Oxid. Med. Cell. Longev..

[32] Hooper C., De Souto Barreto P., Payoux P., Salabert A.S., Guyonnet S., Andrieu S., Sourdet S., Delrieu J., Vellas B. (2017). Association of cortical β-amyloid with erythrocyte membrane monounsaturated and saturated fatty acids in older adults at risk of dementia. J. Nutr. Health Aging.

[33] Praticò D. (2002). Lipid peroxidation and the aging process. Sci. Aging Knowl. Environ..

[34] Sinem F., Dildar K., Gökhan E., Melda B., Orhan Y., Filiz M. (2010). The serum protein and lipid oxidation marker levels in Alzheimer’s disease and effects of cholinesterase inhibitors and antipsychotic drugs therapy. Curr. Alzheimer Res..

[35] Miyazawa T., Suzuki T., Fujimoto K., Kinoshita M. (1996). Age-related change of phosphatidylcholine hydroperoxide and phosphatidylethanolamine hydroperoxide levels in normal human red blood cells. Mech. Ageing Dev..

[36] Choi J.H., Yu B.P. (1995). Brain synaptosomal aging: Free radicals and membrane fluidity. Free Radic. Biol. Med..

[37] Ou T., Yamakawa-Kobayashi K., Arinami T., Amemiya H., Fujiwara H., Kawata K., Saito M., Kikuchi S., Noguchi Y., Sugishita Y. (1998). Methylenetetrahydrofolate reductase and apolipoprotein E polymorphisms are independent risk factors for coronary heart disease in Japanese: A case-control study. Atherosclerosis.

[38] Davignon J., Gregg R.E., Sing C.F. (1988). Apolipoprotein E polymorphism and atherosclerosis. Arteriosclerosis.

[39] Song Y., Stampfer M.J., Liu S. (2004). Meta-analysis: apolipoprotein E genotypes and risk for coronary heart disease. Ann. Intern. Med..

[40] Tammi A., Rönnemaa T., Rask-Nissilä L., Miettinen T.A., Gylling H., Valsta L., Viikari J., Välimäki I., Simell O. (2001). Apolipoprotein E Phenotype Regulates Cholesterol Absorption in Healthy 13-Month-Old Children—The STRIP Study. Pediatr. Res..

[41] Tammi A., Rönnemaa T., Viikari J., Jokinen E., Lapinleimu H., Ehnholm C., Simell O. (2000). Apolipoprotein E4 phenotype increases non-fasting serum triglyceride concentration in infants—The STRIP study. Atherosclerosis.

[42] Verghese P.B., Castellano J.M., Holtzman D.M. (2011). Apolipoprotein E in Alzheimer’s disease and other neurological disorders. Lancet Neurol..

[43] Maiti T.K., Konar S., Bir S., Kalakoti P., Bollam P., Nanda A. (2015). Role of apolipoprotein E polymorphism as a prognostic marker in traumatic brain injury and neurodegenerative disease: A critical review. Neurosurg. Focus.

[44] Maurya P.K., Kumar P., Chandra P. (2015). Biomarkers of oxidative stress in erythrocytes as a function of human age. World J. Methodol..

[45] Jayakumar R., Kusiak J.W., Chrest F.J., Demehin A.A., Murali J., Wersto R.P., Nagababu E., Ravi L., Rifkind J.M. (2003). Red cell perturbations by amyloid beta-protein. Biochim. Biophys. Acta.

[46] Head D., Bugg J.M., Goate A.M., Fagan A.M., Mintun M.A., Benzinger T., Holtzman D.M., Morris J.C. (2012). Exercise Engagement as a Moderator of the Effects of APOE Genotype on Amyloid Deposition. Arch. Neurol..

[47] Nakamura T., Kawarabayashi T., Seino Y., Hirohata M., Nakahata N., Narita S., Itoh K., Nakaji S., Shoji M. (2018). Aging and APOE-ε4 are determinative factors of plasma Aβ42 levels. Ann. Clin. Transl. Neurol..

[48] Frederiksen K.S., Madsen K., Andersen B.B., Beyer N., Garde E., Høgh P., Waldemar G., Hasselbalch S.G., Law I. (2015). Effect of moderate-high intensity aerobic exercise on beta-amyloid accumulation measured with 11C-PiB-PET in patients with mild to moderate Alzheimer’s disease. Alzheimers Dement..

[49] Stillman C.M., Lopez O.L., Becker J.T., Kuller L.H., Mehta P.D., Tracy R.P., Erickson K.I. (2017). Physical activity predicts reduced plasma β amyloid in the Cardiovascular Health Study. Ann. Clin. Transl. Neurol..

[50] Brown B.M., Peiffer J.J., Taddei K., Lui J.K., Laws S.M., Gupta V.B., Taddei T., Ward V.K., Rodrigues M.A., Burnham S. (2013). Physical activity and amyloid-β plasma and brain levels: Results from the Australian Imaging, Biomarkers and Lifestyle Study of Ageing. Mol. Psychiatry.

[51] Kobayashi Y., Nakatsuji A., Aoi W., Wada S., Kuwahata M., Kido Y. (2014). Intense exercise increases protein oxidation in spleen and liver of mice. Nutr. Metab. Insights.

[52] Gawron-Skarbek A., Chrzczanowicz J., Kostka J., Nowak D., Drygas W., Jegier A., Kostka T. (2015). Physical Activity, Aerobic Capacity, and Total Antioxidant Capacity in Healthy Men and in Men with Coronary Heart Disease. Oxid. Med. Cell. Longev..

[53] Gustaw-Rothenberg K., Kowalczuk K., Stryjecka-Zimmer M. (2010). Lipids’ peroxidation markers in Alzheimer’s disease and vascular dementia. Geriatr. Gerontol. Int..

[54] Prasinou P., Dafnis I., Giacometti G., Ferreri C., Chroni A., Chatgilialoglu C. (2017). Fatty acid-based lipidomics and membrane remodeling induced by apoE3 and apoE4 in human neuroblastoma cells. Biochim. Biophys. Acta Biomembr..

[55] Parkhouse W.S., Willis P.E., Zhang J. (1995). Hepatic lipid peroxidation and antioxidant enzyme responses to long-term voluntary physical activity and aging. AGE.

[56] Nicolle C., Cardinault N., Gueux E., Jaffrelo L., Rock E., Mazur A., Amouroux P., Rémésy C. (2004). Health effect of vegetable-based diet: Lettuce consumption improves cholesterol metabolism and antioxidant status in the rat. Clin. Nutr..

[57] Jiang H., Wang Z., Ma Y., Qu Y., Lu X., Luo H. (2015). Effects of Dietary Lycopene Supplementation on Plasma Lipid Profile, Lipid Peroxidation and Antioxidant Defense System in Feedlot Bamei Lamb. Asian-Australas J. Anim. Sci..

[58] Van der Veen J.N., Kennelly J.P., Wan S., Vance J.E., Vance D.E., Jacobs R.L. (2017). The critical role of phosphatidylcholine and phosphatidylethanolamine metabolism in health and disease. Biochim. Biophys. Acta Biomembr..

[59] Cao J., Gaamouch F.E., Meabon J.S., Meeker K.D., Zhu L., Zhong M.B., Bendik J., Elder G., Jing P., Xia J. (2017). ApoE4-associated phospholipid dysregulation contributes to development of Tau hyper-phosphorylation after traumatic brain injury. Sci. Rep..

[60] Sumikawa K., Mu Z., Inoue T., Okochi T., Yoshida T., Adachi K. (1993). Changes in erythrocyte membrane phospholipid composition induced by physical training and physical exercise. Eur. J. Appl. Physiol. Occup. Physiol..

[61] Saha S.S., Chakraborty A., Ghosh S., Ghosh M. (2012). Comparative study of hypocholesterolemic and hypolipidemic effects of conjugated linolenic acid isomers against induced biochemical perturbations and aberration in erythrocyte membrane fluidity. Eur. J. Nutr..

[62] Cazzola R., Russo-Volpe S., Cervato G., Cestaro B. (2003). Biochemical assessments of oxidative stress, erythrocyte membrane fluidity and antioxidant status in professional soccer players and non-active controls. Eur. J. Clin. Investig..

[63] Kaestner L., Bogdanova A. (2014). Regulation of red cell life-span, erythropoiesis, senescence, and clearance. Front. Physiol..

